# Differential Expression Profiles and Functional Prediction of circRNAs in Necrotizing Enterocolitis

**DOI:** 10.1155/2021/9862066

**Published:** 2021-11-03

**Authors:** Ya Pan, Wenjuan Chen, Xiangyun Yan, Boshi Yu, Shuwen Yao, Xiaohui Chen, Shuping Han

**Affiliations:** Department of Pediatrics, Women's Hospital of Nanjing Medical University, Nanjing Maternity and Child Health Care Hospital, Nanjing, Jiangsu, China

## Abstract

Circular RNAs (circRNAs), a novel type of noncoding RNAs, have been demonstrated to behave as microRNA (miRNA) sponges to exert their effects during pathological processes of diseases. However, the roles of circRNAs have not been explored in necrotizing enterocolitis (NEC). This study sought to identify differentially expressed circRNAs and predict their potential biological functions in NEC. circRNA expression profiles in terminal ileum from newborn rats with NEC and normal controls were explored using next-generation sequencing. In the NEC group, 53 circRNAs were significantly differentially expressed, including 9 upregulated and 44 downregulated. Gene ontology and Kyoto Encyclopedia of Genes and Genomes pathway analyses were conducted, and circRNA-miRNA interaction networks were generated to predict the potential roles of circRNAs in NEC progression. Further investigation revealed that most circRNAs include miRNA binding sites and that some are implicated in NEC development. In conclusion, this study's findings demonstrate that differentially expressed circRNAs are involved in NEC development via their interactions with miRNAs, making them prospective targets for NEC diagnosis and treatment.

## 1. Introduction

Necrotizing enterocolitis (NEC) is a devastating gastrointestinal disease in neonates that mainly affects preterm infants. Although significant advances have been made in neonatal care in recent decades, NEC is associated with high morbidity and mortality rates [1]. A previous systematic review reported that approximately 7% of infants born with deficient birth weight admitted to NICU have NEC [2]. In addition, surviving infants exhibit digestive tract and neurological sequelae [3]. NEC pathogenesis is multifactorial, including premature delivery, dysregulation of proinflammatory factors, and bacterial colonization [4]. Although NEC pathogenesis has been extensively explored, the pathophysiological mechanism of NEC remains unclear, and effective therapeutic methods are now unavailable.

Circular RNAs (circRNAs) are a class of endogenous noncoding RNAs characterized by covalently closed-loop structures but lack 5′ to 3′ polarity and a polyadenylated tail [5]. circRNAs are mainly derived from the exons of protein-coding genes via “back splicing” and exhibit cell-type or tissue-specific expression [5]. Recent studies report that circRNAs are expressed in most human cells and serve a critical role in regulating gene expression at the posttranscriptional level [6]. circRNAs have been linked to certain diseases, such as diabetic colonic dysmotility [7], inflammatory bowel disease [8], sepsis [9], innate colitis [10], and colorectal cancer [11]. For instance, circHIPK3 was proved to be a major regulator of intestinal epithelial repair following acute injury [12]. In sepsis rats, circDMNT3B downregulation resulted in dysfunction of intestinal mucosal permeability via sponging miR-20b-5p [13]. However, the role of circRNAs in NEC remains unknown.

In this study, next-generation sequencing (NGS) was performed to identify expression profiles of circRNAs in the terminal ileum from NEC mice and normal controls. The findings of this investigation indicate differential expression of circRNAs between the two groups. Bioinformatics analysis revealed that differentially expressed circRNAs contribute significantly to initiation and progression of NEC through their interaction with NEC-related miRNAs.

## 2. Materials and Methods

### 2.1. Ethics Statement

All experiments in our study were approved by the Animal Ethics Committee of Nanjing Medical University. The study was performed following the guidelines by the Animal Protection Law and the Care and Use of Laboratory Animals of the National Research Council.

### 2.2. Establishment of NEC Model

Pregnant experimental SD rats were purchased from the Experimental Animal Center of Nanjing Medical University. NEC procedure was performed on neonatal rats as described previously [14]. Neonatal rats in the NEC group were fed by gavage high osmotic pressure formula milk (Similac Advance infant formula (Ross Pediatrics)/Esbilac canine milk replacer, 2 : 1) three times per day. Animals were subsequently subjected to hypoxic environment stimulation (5% O_2_, 95% N_2_) for 5 min, three times daily for four days.

NEC mainly occurs in the distal ileum. A 3 cm sample of the distal ileum was obtained and was divided into two parts. A 2 cm sample of the most distal ileum was employed for circular RNA sequencing, whereas the remaining 1 cm was utilized for H&E staining to evaluate NEC model. Pathological scoring of ileal injuries was performed as follows [15]: 0 point (normal): intestinal mucosa villi intact; 1 point (mild damage): the villi are slightly edematous and only the top of the villi is shed; 2 points (moderate damage): moderate necrosis; 3 points (severe injury): loss of villi but still recognizable crypt; and 4 points (necrosis): absence of mucosal epithelium or necrosis. The extent of intestinal damage was determined based on the highest injury score observed in the section. NEC is defined when a histology score is equal to or higher than 2 points. The fresh villus was taken and immediately frozen in liquid nitrogen and then washed several times with PBS. The samples were then stored in liquid nitrogen until subsequent use.

### 2.3. RNA Extraction

Total RNA was extracted from the samples using TRIzol Reagent (Invitrogen, Carlsbad CA, USA), according to the manufacturer's instructions. RNAprep Pure Tissue Kit (TIANGEN, DP431) was used for subsequent RNA preparation. Based on the concentration of each sample, 1000 ng total RNA was added into 20 *μ*L reverse transcription reaction. RNA concentration was determined by OD260/OD280 using a NanoDrop ND-1000 spectrophotometer. RNA's integrity was assessed using electrophoresis on a denaturing agarose gel.

### 2.4. Detection and Determination of circRNAs

RNA sequencing and determination were performed using Decode Genomics Biotechnology Co, Ltd (Nanjing, Jiangsu, China). In summary, whole terminal ileum tissues were sequenced. Total RNA was isolated using the standard TRIzol (Invitrogen) protocol. Genomic DNA was removed using DNase treatment. Total RNA was subjected to ribosomal RNA depletion following the manufacturer's protocol in the RiboMinus kit (Life Technology). The quality of RNA and absence of contaminating ribosomal RNA were confirmed using Agilent 2100 Bioanalyzer. Strand-specific libraries were constructed following the manufacturer's instructions (Illumina), and each library was loaded into one lane of Illumina HiSeq 2500 for 2 × 150 bp pair-end sequencing. circRNA prediction of sequencing results was performed using CIRI algorithm.

### 2.5. Bioinformatics Analyses

Expression profiles of circRNAs in the terminal ileum were analyzed, and differentially expressed circRNAs were screened using edgeR. Scatter plot filtering and volcano plot filtering were utilized to assess variations between NEC cases and normal controls. Differentially expressed circRNAs were identified (*P* < 0.05). Parental gene functions of these differential circRNAs were analyzed using DAVID Bioinformatics Resources 6.8 tool (https://david.ncifcrf.gov/home.jsp). Gene ontology (GO) analysis of parental genes was performed on three terms, namely, biological processes (BP), cellular components (CC), and molecular functions (MF). In addition, related biological pathways were analyzed using Kyoto Encyclopedia of Genes and Genomes (KEGG). For functional analysis, –log10 (*P* value) was used as enrichment score indicating the significance of correlation, and the number of target genes was determined.

### 2.6. Quantitative Real-Time Polymerase Chain Reaction (qRT-PCR) Analysis

cDNA synthesis was performed on each sample using reverse transcription with random primers following TaKaRa PrimeScript™ RT Master Mix Kit instructions. SYBR green was used for qRT-PCR to evaluate chip results. Experimental data were analyzed using the 2^-*ΔΔ*CT^ method. All data were presented as the average of three independent experiments.

### 2.7. Annotation for circRNA/miRNA Interaction

Interactions between circRNAs/miRNAs were predicted using miRanda tool (http://www.microrna.org/microrna/home.do/). miRNA response elements (MREs) on circRNAs were searched from the database. miRNAs were then selected based on seed-match sequences. A graph of circRNA/miRNA network was constructed based on the correlation analysis between differential circRNAs and miRNAs using Cytoscape 3.01.

### 2.8. Statistical Analyses

Statistical analyses were performed using Statistical Program for Social Sciences Version 22 (SPSS.22) and GraphPad prism 8.0. All data were presented as mean ± SD for triplicate independent measurements. The Student *t*-test was employed to compare differences between experimental groups. Differences with *P* values less than 0.05 (*P* < 0.05) were considered statistically significant.

## 3. Results

### 3.1. Establishment and Validation of NEC Rat Model

**Figure 1:**
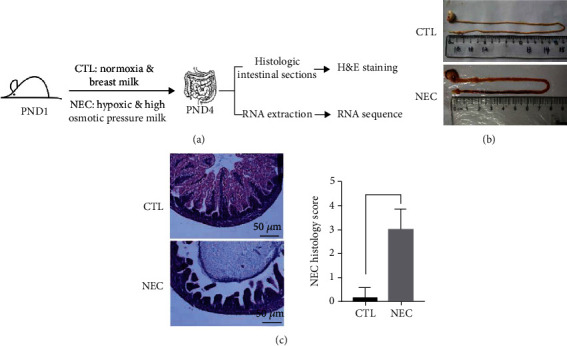
Establishment and validation of the NEC model: (a) experimental design; (b) intestinal morphology of neonatal rats that were either mother-fed or induced to develop NEC; (c) representative H&E micrographs proximal to ileocecal valve and pathological scores (^∗^*P* < 0.05).

A NEC rat model was established to explore circRNA expression profiles (Figure 1(a)). The NEC group rat presented symptoms such as diarrhea, abdominal distension, watery stools, and respiratory distress. The control group rats did not present with these symptoms. Necropsy was performed on day 4 of NEC modeling. The intestines of the NEC group rats exhibited a black appearance with signs of luminal flatulence (Figure 1(b)). Analysis of H&E staining revealed submucosal edema, villus core separation, and sloughing of epithelium for animals in the NEC group. Pathological changes in intestinal architecture were determined based on NEC scoring system. The pathological score of the NEC group was significantly higher compared with that of the control group (Figure 1(c)), indicating that the NEC animal model was successfully established.

### 3.2. Identification of Differentially Expressed circRNAs in the NEC Model

**Figure 2:**
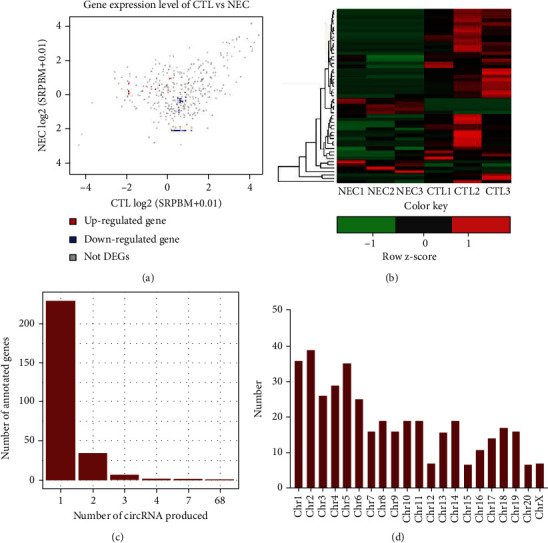
Identification of differentially expressed circRNAs in the NEC model. (a) Scatter plot of differentially expressed circRNAs. (b) Heatmap of differentially expressed miRNAs. (c) The number of circRNAs generated by host genes. Some linear genes produce only one circularized RNA, whereas some linear genes produce multiple circularized RNAs. The abscissa indicates the number of circRNAs produced by linear RNAs, whereas the ordinate is the corresponding number of linear RNAs. (d) Distribution of circRNAs in the chromosome. SRPBM: spliced reads per billion mapped.

circRNA expression profiles in terminal ileum tissues of the NEC group were explored using NGS technology. Differentially expressed circRNAs between two groups were used to generate a scatter plot. A total of 53 differentially expressed circRNAs between normal and NEC groups were identified (*P* < 0.05), including 9 upregulated and 44 downregulated circRNAs (Figure 2(a)). A heat map was employed to present changes in expression levels of circRNAs of different groups (Figure 2(b)). Although previous studies reported that most host genes could only produce a single circRNA, the findings of this study reveal that some genes produced up to four or five kinds of hypotype circRNAs (Figure 2(c)). Moreover, the analysis demonstrated that host genes of circRNAs are distributed in each chromosome (Figure 2(d)). The significantly differentially expressed circRNAs are presented in Supplementary Table 1.

### 3.3. GO and KEGG Pathway Analysis

**Figure 3:**
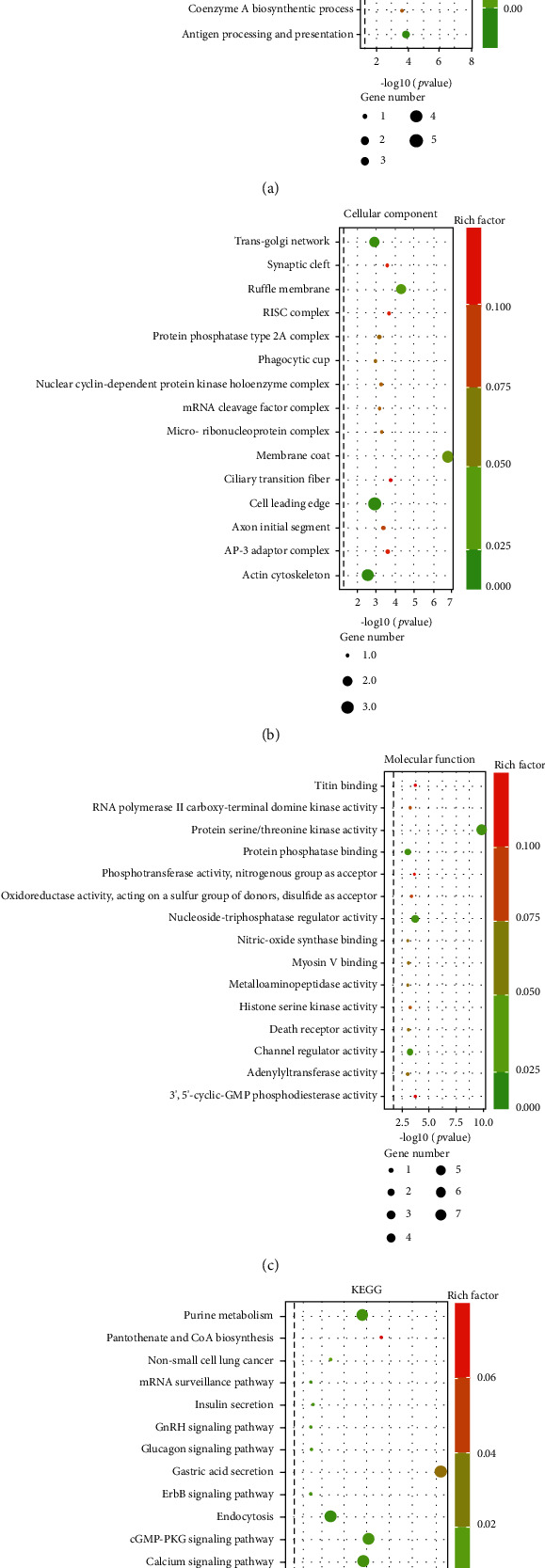
GO and pathway analysis of differentially expressed circRNAs: (a) biological process category; (b) cellular component category; (c) molecular function category; (d) canonical signaling pathways.

The function of circRNAs is similar to that of their host genes. GO and KEGG pathway analyses were used to further explore host genes for differentially expressed circRNAs. GO analysis revealed that these genes were significantly enriched in “regulation of extrinsic apoptotic signaling pathway” (biological process; Figure 3(a)), “ciliary transition fiber” (cellular component; Figure 3(b)), and “death receptor activity” (molecular function; Figure 3(c)). KEGG analysis results indicated that these genes were significantly enriched in gastric acid secretion, pantothenate and CoA biosynthesis, and cGMP-PKG signaling pathway (Figure 3(d)).

### 3.4. Validation of Differentially Expressed circRNAs

**Figure 4:**
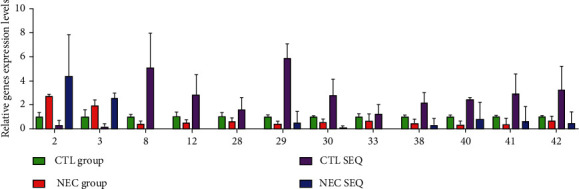
The expression levels of 12 circRNAs validated using qRT-PCR.

qRT-PCR was utilized to evaluate the reliability of NGS results. A total of 12 differentially expressed circRNAs were randomly selected from the same sample used for NGS analysis and employed for qRT-PCR. Analysis of qRT-PCR results revealed that the expression profiles of these circRNAs were consistent with NGS results (Figure 4). This implies that NGS data are reliable. Primer sequences used are shown in Supplementary Table 2.

### 3.5. The Detailed Annotation for the Interaction between circRNAs and miRNAs

**Figure 5:**
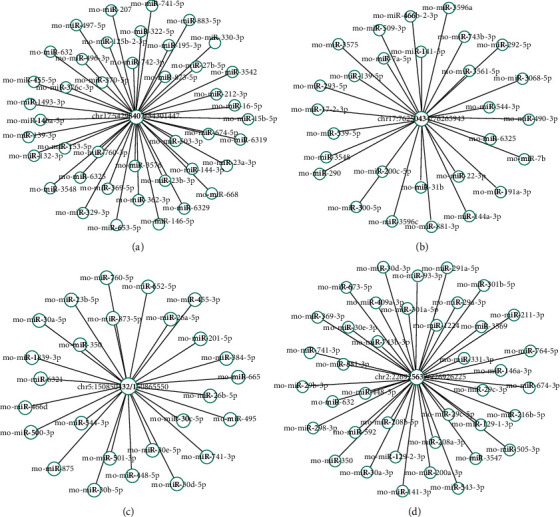
Network of the relationship between circRNAs and miRNAs. circRNA-miRNA coexpression network was constructed using Cytoscape V3.6.1 software. The nodes at the circle center represent circRNA, whereas the nodes outside the circle represent miRNA.

Previous studies reported that circRNAs perform various biological functions through downstream miRNAs. To explore target miRNAs of circRNAs, target scanning and Miranda databases were used to theoretically predict miRNAs based on conservative seed-matching sequences. The relationship between circRNA and miRNA was presented as an interaction network (the top four differentially expressed circRNAs) (Figure 5).

## 4. Discussion

NEC is a complex multifactorial disease, and its exact mechanism has not been fully elucidated [16]. Recent studies indicate that circRNAs regulate numerous important cellular physiological activities, including cell differentiation, proliferation, and apoptosis through cis- or transregulation [17]. However, the role of circRNAs in NEC remains unknown. In this study, NGS technology was employed to investigate expression profiles of circRNAs with significant differences associated with NEC. Host genes for differentially expressed circRNAs were then analyzed using bioinformatics tools. Additionally, circRNA-miRNA interaction networks were established based on predictions results. This study investigated circRNA expression levels of NEC and CTL groups using deep Illumina sequencing. A total of 53 differentially expressed circRNAs were identified. This finding implies that NEC and CTL groups have distinct expression profiles of these circRNAs. Biological functions and potential pathways linked to host genes for differentially expressed circRNAs were predicted using GO and KEGG pathway analyses. GO analysis identifies host genes, implicated in BP, CC, and MF and associated with development of diseases. GO analysis results indicated that host genes for differentially expressed circRNAs are correlated with regulating extrinsic apoptotic signaling pathways, ciliary transition fiber, and death receptor activity, all of which are implicated in NEC development. Apoptosis of intestinal epithelial cells is linked to NEC progression [18]. Extrinsic lipopolysaccharide-induced reactive oxygen species accumulation results in NF-*κ*B activation and subsequent release of proinflammation cytokines, including IL-6 and IL-1*β*, which are involved in NEC pathogenesis [19, 20]. KEGG pathway analysis identified pathways that are mainly linked to NEC progression. cGMP-PKG signaling pathway activation suppresses Wnt/*β*-catenin signaling, which has been linked to attenuation of intestinal injury in NEC in experimental animals [21, 22].

Recent studies indicate that circRNAs function as miRNA sponges and regulate downstream miRNAs involved in various pathological mechanisms. For instance, circPKNOX1 influences progression of intervertebral disc disease by regulating KIAA0355 expression via miR-370-3p [23]. Downregulation of hsa_circ_0002874 regulates the miR1273f/MDM2/P53 signaling pathway to reverse paclitaxel resistance of non-small-cell lung cancer and induces apoptosis *in vitro* and *in vivo* [24]. hsa_circ_0107593 exhibits tumor-suppressing activity in cervical cancer by physically binding to hsa-miR-20a-5p, hsa-miR-93-5p, and hsa-miR-106b-5p [25]. In this study, biological prediction analysis revealed that differentially expressed circRNAs might bind to various miRNAs. Notably, the identified miRNAs are implicated in regulating physiological and pathological processes of NEC. For instance, the interaction between chr17:54298407|54301447 and miR-146b-5p, miR-674-5p, and miR-212-3p was observed. Previous research indicates that miR-146b-5p is critical for maintaining intestinal mucosal homeostasis [26, 27]. Inhibiting miR-674-5p attenuates intestinal inflammation and promotes intestinal regeneration, protecting mice from endotoxemia-induced intestinal injury [28]. miR-212-3p directly targets HMGB1 to suppress inflammatory response [29]. In addition, chr17:76250434|76265943 interacted with miR-139-5p and miR-3068-5p. miR-139-5p has been implicated in maintaining intestinal homeostasis and protection against colitis *in vivo* [30]. Inhibition of miR-3068-5p represses p65 phosphorylation and reduces NLRP3 inflammasome and IL-1*β* and IL-18 secretion [10]. These findings indicate that dysregulated circRNAs may interact with these miRNAs, thus promoting NEC development.

Notably, this study had a few limitations. First, the sample size was small, implying that further studies should be conducted using larger sample sizes to avoid bias caused by individual differences. In addition, this study only used intestinal tissues as experimental materials, demonstrating that future studies should include more biological samples associated with NEC, including faeces or blood plasma. Moreover, dysregulated circRNAs in rats at only one stage of NEC were explored, indicating that further studies should explore dysregulated circRNAs in the intestine of rats with different NEC degrees to identify more targets for treatment. Moreover, intestinal macrophages will be performed in our future studies to provide new insight into NEC pathogenesis.

In conclusion, circRNAs reveal significantly higher expression levels in intestinal tissues of NEC group rats than the levels in control group rats. The findings of this study indicate that circRNAs significantly contribute to NEC occurrence. This work provides a basis for elucidating the pathophysiological mechanism underlying intestinal changes following NEC and offers new approaches for diagnosing and treating NEC.

## Data Availability

All data that support the findings of this study are available from the corresponding author upon reasonable request.

## Supplementary Materials

Supplementary 1 Supplementary Table 1: a list of differentially expressed circRNAs.

Supplementary 2 Supplementary Table 2: list of primer sequences used in the study.
