# Supplementary material for: Differential Expression Profiles and Functional Prediction of circRNAs in Necrotizing Enterocolitis
Source: Biomed Res Int. 2021 Nov 3;2021:9862066. doi: 10.1155/2021/9862066 (PMC8581514; doi:10.1155/2021/9862066)
Supplement: Supplementary 1 — Supplementary Table 1: a list of differentially expressed circRNAs. [file 9862066.f1.docx]

| GeneID | NEC-1 | NEC-2 | NEC-3 | CTL-1 | CTL-2 | CTL-3 | logFC | P value | chr | Host_gene_id |  |  |
| --- | --- | --- | --- | --- | --- | --- | --- | --- | --- | --- | --- | --- |
| chr10:86175962\|86195240 | 0 | 0 | 2.087171 | 6.081222 | 2.648761 | 1.521017 | -4.285013 | 0.047187 | chr10 | ENSRNOG00000006000 |  |  |
| chr6:22033342\|22038870 | 0 | 4.997364 | 0 | 4.263125 | 6.732267 | 5.139989 | -4.568307 | 0.046843 | chr6 | ENSRNOG00000027191 |  |  |
| chr8:23508992\|23560371 | 0 | 1.612053 | 0 | 1.504632 | 5.352704 | 2.832239 | -4.271702 | 0.044298 | chr8 | ENSRNOG00000056108 |  |  |
| chr5:144539229\|144547479 | 0 | 0 | 2.030761 | 2.382334 | 4.745696 | 1.783262 | -4.420491 | 0.039281 | chr5 | ENSRNOG00000025596 |  |  |
| chr14:10786399\|10794860 | 2.444993 | 0 | 0 | 2.50772 | 2.538396 | 2.150404 | -4.440356 | 0.038847 | chr14 | ENSRNOG00000002203 |  |  |
| chr2:20507150\|20531444 | 2.078244 | 0 | 0 | 2.194255 | 1.54511 | 3.514074 | -4.442469 | 0.038333 | chr2 | ENSRNOG00000016213 |  |  |
| chr5:137096509\|137112930 | 0 | 0 | 1.241021 | 0.689623 | 6.235624 | 2.569995 | -4.625069 | 0.03098 | chr5 | n/a | |  |
| chr10:94083626\|94187819 | 16.01471 | 9.349906 | 2.989732 | 14.41939 | 54.07886 | 35.24564 | -1.831557 | 0.027801 | chr10 | n/a | |  |
| chr5:128083027\|128083901 | 2.200494 | 9.242436 | 1.748711 | 0.877702 | 0 | 0 | 4.60947 | 0.026072 | chr5 | n/a | |  |
| chr9:20596863\|20599410 | 0.977997 | 0 | 0 | 3.13465 | 1.76584 | 1.625915 | -4.537752 | 0.023713 | chr9 | ENSRNOG00000011517 |  |  |
| chr3:160491308\|160495240 | 0 | 1.450848 | 0 | 4.012353 | 4.359419 | 3.356728 | -4.609265 | 0.022692 | chr3 | ENSRNOG00000013529 |  |  |
| chr8:117318623\|117334586 | 0 | 0 | 1.410251 | 1.316553 | 3.531681 | 4.930194 | -4.775768 | 0.021134 | chr8 | ENSRNOG00000025071 |  |  |
| chr6:129864989\|129890111 | 0 | 0 | 0 | 0.125386 | 1.986571 | 1.730813 | -4.807628 | 0.018622 | chr6 | ENSRNOG00000005274 |  |  |
| chr6:72418120\|72430205 | 1.650371 | 0 | 0 | 2.319641 | 4.580149 | 1.888159 | -4.855056 | 0.018346 | chr6 | ENSRNOG00000060335 |  |  |
| chr6:106053312\|106055105 | 2.078244 | 3.116635 | 2.482041 | 0.564237 | 0 | 0 | 4.820877 | 0.016444 | chr6 | ENSRNOG00000007646 |  |  |
| chr7:140828965\|140855908 | 0 | 0 | 0 | 0.125386 | 2.703943 | 1.783262 | -4.96471 | 0.015847 | chr7 | ENSRNOG00000057315 |  |  |
| chr14:82168512\|82171569 | 0 | 0 | 2.538452 | 8.150091 | 4.414601 | 5.192438 | -5.221602 | 0.014506 | chr14 | n/a | |  |
| chr11:69018396\|69064671 | 0 | 0 | 0 | 0.376158 | 1.931388 | 1.311222 | -5.080976 | 0.010465 | chr11 | ENSRNOG00000002215 |  |  |
| chr16:23555919\|23600154 | 0 | 0 | 1.353841 | 4.388511 | 4.690514 | 3.409177 | -5.144721 | 0.009404 | chr16 | ENSRNOG00000013884 |  |  |
| chr7:116421054\|116558126 | 4.584363 | 6.985562 | 20.08197 | 0 | 2.869491 | 0 | 6.507911 | 0.006742 | chr7 | n/a | |  |
| chr7:60397574\|60408823 | 0 | 0 | 0 | 1.003088 | 1.158833 | 1.468568 | -5.29418 | 0.006581 | chr7 | ENSRNOG00000005927 |  |  |
| chr8:130762665\|130768360 | 0.305624 | 0 | 0 | 3.886967 | 1.32438 | 3.146932 | -5.035613 | 0.005954 | chr8 | ENSRNOG00000004050 |  |  |
| chr3:8887351\|8892631 | 0 | 0 | 1.579481 | 5.516985 | 4.856061 | 7.185495 | -5.555629 | 0.005706 | chr3 | ENSRNOG00000025185 |  |  |
| chr1:126573474\|126583015 | 5.073361 | 3.170371 | 5.189723 | 0 | 2.096936 | 0 | 5.774826 | 0.00514 | chr1 | n/a | |  |
| chr1:91883192\|91887419 | 0.855748 | 1.558318 | 1.635891 | 0 | 0 | 0 | 5.634746 | 0.004876 | chr1 | ENSRNOG00000052814 |  |  |
| GeneID | NEC-1 | NEC-2 | NEC-3 | CTL-1 | CTL-2 | CTL-3 | logFC | P value | chr | Host_gene_id |  |  |
| chr3:13270114\|13288615 | 0.427874 | 0 | 0 | 1.818097 | 2.924673 | 3.461625 | -5.246147 | 0.00435 | chr3 | ENSRNOG00000022162 |  |  |
| chr14:10786399\|10787114 | 0 | 0 | 0 | 1.692711 | 2.262483 | 0.786733 | -5.539227 | 0.004049 | chr14 | ENSRNOG00000002203 |  |  |
| chr2:128562842\|128590589 | 1.039122 | 1.719523 | 1.523071 | 0 | 0 | 0 | 5.733752 | 0.003873 | chr2 | ENSRNOG00000014139 |  |  |
| chr1:20022792\|20047889 | 0 | 0 | 0 | 0.438851 | 2.262483 | 2.832239 | -5.672861 | 0.003854 | chr1 | n/a | |  |
| chr10:85164768\|85172858 | 0 | 0 | 0 | 0.752316 | 2.041753 | 2.150404 | -5.684907 | 0.003083 | chr10 | ENSRNOG00000023095 |  |  |
| chr6:871525\|907083 | 0 | 0 | 0 | 0.376158 | 2.814308 | 3.409177 | -5.866382 | 0.002892 | chr6 | ENSRNOG00000004208 |  |  |
| chr7:62899716\|62931338 | 0 | 0 | 0 | 0.564237 | 2.979856 | 2.517546 | -5.895787 | 0.002231 | chr7 | ENSRNOG00000042668 |  |  |
| chr8:87268950\|87273285 | 0 | 0 | 0 | 0.752316 | 1.986571 | 3.356728 | -5.921886 | 0.001879 | chr8 | ENSRNOG00000011521 |  |  |
| chr2:227687641\|227702229 | 0 | 0 | 0 | 1.191167 | 2.593578 | 1.678364 | -5.867426 | 0.001775 | chr2 | ENSRNOG00000015353 |  |  |
| chr6:28022406\|28084649 | 2.200494 | 1.343377 | 1.861531 | 0 | 0 | 0 | 6.114335 | 0.001476 | chr6 | ENSRNOG00000011914 |  |  |
| chr17:82598585\|82646490 | 0 | 0 | 0 | 2.445027 | 2.648761 | 1.311222 | -5.980223 | 0.001147 | chr17 | ENSRNOG00000052916 |  |  |
| chr19:37135068\|37156898 | 0 | 0 | 0 | 0.564237 | 2.924673 | 4.982643 | -6.288902 | 0.001029 | chr19 | ENSRNOG00000014647 |  |  |
| chr14:81267948\|81280976 | 2.567243 | 1.719523 | 1.635891 | 0 | 0 | 0 | 6.237587 | 0.001026 | chr14 | ENSRNOG00000011847 |  |  |
| chr13:35597462\|35608096 | 0 | 0 | 0 | 1.128474 | 2.869491 | 2.674892 | -6.16082 | 0.00087 | chr13 | ENSRNOG00000002538 |  |  |
| chr13:70951556\|70976184 | 0 | 0 | 0 | 1.316553 | 3.421316 | 2.045506 | -6.169232 | 0.000822 | chr13 | ENSRNOG00000002775 |  |  |
| chr2:231042213\|231078948 | 0 | 0 | 0 | 1.191167 | 3.035038 | 2.674892 | -6.211182 | 0.000752 | chr2 | ENSRNOG00000011589 |  |  |
| chr1:165646261\|165654319 | 0 | 0 | 0 | 1.065781 | 2.262483 | 4.615501 | -6.298719 | 0.000703 | chr1 | ENSRNOG00000018176 |  |  |
| chr2:236645209\|236651429 | 0 | 0 | 0 | 1.441939 | 3.200586 | 2.30775 | -6.223208 | 0.000677 | chr2 | ENSRNOG00000011311 |  |  |
| chr2:24142433\|24161334 | 0 | 0 | 0 | 1.88079 | 3.035038 | 2.045506 | -6.210645 | 0.000637 | chr2 | ENSRNOG00000010624 |  |  |
| chr18:36244090\|36271261 | 0 | 0 | 0 | 0.752316 | 3.807593 | 4.510603 | -6.483349 | 0.00055 | chr18 | ENSRNOG00000054049 |  |  |
| chr19:32470120\|32497560 | 0 | 0 | 0 | 1.818097 | 2.096936 | 4.19591 | -6.369148 | 0.000464 | chr19 | ENSRNOG00000011697 |  |  |
| chr1:253120469\|253134791 | 0 | 0 | 0 | 1.88079 | 4.745696 | 1.783262 | -6.397938 | 0.000454 | chr1 | ENSRNOG00000018944 |  |  |
| chr3:161474539\|161487731 | 0 | 0 | 0 | 4.952748 | 8.056647 | 1.206324 | -6.811602 | 0.000282 | chr3 | ENSRNOG00000017824 |  |  |
| chr13:68752167\|68763048 | 0 | 0 | 0 | 1.630018 | 7.725552 | 2.360199 | -6.783772 | 0.000225 | chr13 | ENSRNOG00000032258 |  |  |
| chr2:226925637\|226926225 | 0 | 0 | 0 | 1.692711 | 3.090221 | 12.58773 | -7.154428 | 0.000155 | chr2 | ENSRNOG00000014443 |  |  |
| GeneID | NEC-1 | NEC-2 | NEC-3 | CTL-1 | CTL-2 | CTL-3 | logFC | P value | chr | Host_gene_id |  |  |
| chr5:150850432\|150865550 | 0 | 0 | 0 | 4.701976 | 8.77402 | 1.678364 | -7.018082 | 0.000151 | chr5 | ENSRNOG00000010396 |  |  |
| chr17:76250434\|76265943 | 9.046475 | 1.397112 | 5.753823 | 0 | 0 | 0 | 7.446198 | 8.38E-05 | chr17 | ENSRNOG00000023593 |  |  |
| chr17:54298407\|54301447 | 0 | 0 | 0 | 7.021617 | 10.65023 | 15.99691 | -8.466727 | 4.98E-06 | chr17 | ENSRNOG00000017791 |  |  |
