# Supplementary material for: Differential Expression Profiles and Functional Prediction of circRNAs in Necrotizing Enterocolitis
Source: Biomed Res Int. 2021 Nov 3;2021:9862066. doi: 10.1155/2021/9862066 (PMC8581514; doi:10.1155/2021/9862066)
Supplement: Supplementary 2 — Supplementary Table 2: list of primer sequences used in the study. [file 9862066.f2.docx]

| circRNA | Number | Primer sequences | Target length |
| --- | --- | --- | --- |
| chr5:128083027\|128083901 | RcircR-2F | GGCAGGTCTCAGGTAT | 89 |
|  | RcircR-2R | ACAGTGCTTCAGGGAT |  |
| chr6:106053312\|106055105 | RcircR-3F | CCACTGAGAACCCGTAT | 244 |
|  | RcircR-3R | GAAGTGCCCAAGGAGA |  |
| chr5:150850432\|150865550 | RcircR-8F | GCGGTCTCAGGCTTCT | 235 |
|  | RcircR-8R | TGTCTACCCTCAGGCAAC |  |
| chr1:253120469\|253134791 | RcircR-12F | CTGGTTTGCCGTTGAA | 129 |
|  | RcircR-12R | TGAAGACCGAGGACTGAT |  |
| chr14:10786399\|10787114 | RcircR-28F | CAGCCTCAATGCTATCC | 176 |
|  | RcircR-28R | CCACAACTCAGCCCTC |  |
| chr3:8887351\|8892631 | RcircR-29F | AGGTAAGCCCTATCATCCA | 126 |
|  | RcircR-29R | GCTGAAGCCTCCTTCTAAT |  |
| chr8:130762665\|130768360 | RcircR-30F | CCTCGCTTGAACCCT | 235 |
|  | RcircR-30R | TTGCGTACTCTGCTCC |  |
| chr11:69018396\|69064671 | RcircR-33F | ACCGTGAGGTTGACTTGG | 197 |
|  | RcircR-33R | TGGAGGTTGTGGAGGGA |  |
| chr9:20596863\|20599410 | RcircR-38F | TCGCAGGCTCGTGTTG | 236 |
|  | RcircR-38R | AAAGGCAGGGCTGAAGA |  |
| chr14:10786399\|10794860 | RcircR-40F | ACAGCCTCAATGCTATCC | 296 |
|  | RcircR-40R | TCAAGCGTCTCCACCC |  |
| chr5:144539229\|144547479 | RcircR-41F | GTTTCCCAACAGTGCC | 91 |
|  | RcircR-41R | ATGTGACAAGACGACCAG |  |
| chr8:23508992\|23560371 | RcircR-42F | AAAGGGTCCATAGGTCAT | 200 |
|  | RcircR-42R | GAAGTTGGGAAGTTTGTCT |  |
